# Predicting Clinical Dementia Rating Using Blood RNA Levels

**DOI:** 10.3390/genes11060706

**Published:** 2020-06-26

**Authors:** Justin B. Miller, John S. K. Kauwe

**Affiliations:** Department of Biology, Brigham Young University, Provo, UT 84602, USA; jmiller@byu.edu

**Keywords:** diagnosis, machine learning, chloride intracellular channel 1 (*CLIC1*), clinical dementia rating, Alzheimer’s disease

## Abstract

The Clinical Dementia Rating (CDR) is commonly used to assess cognitive decline in Alzheimer’s disease patients and is included in the Alzheimer’s Disease Neuroimaging Initiative (ADNI) dataset. We divided 741 ADNI participants with blood microarray data into three groups based on their most recent CDR assessment: cognitive normal (CDR = 0), mild cognitive impairment (CDR = 0.5), and probable Alzheimer’s disease (CDR ≥ 1.0). We then used machine learning to predict cognitive status using only blood RNA levels. Only one probe for chloride intracellular channel 1 (*CLIC1*) was significant after correction. However, by combining individually nonsignificant probes with *p*-values less than 0.1, we averaged 87.87% (s = 1.02) predictive accuracy for classifying the three groups, compared to a 55.46% baseline for this study due to unequal group sizes. The best model had an overall precision of 0.902, recall of 0.895, and a receiver operating characteristic (ROC) curve area of 0.904. Although we identified one significant probe in *CLIC1*, *CLIC1* levels alone were not sufficient to predict dementia status and cannot be used alone in a clinical setting. Additional analyses combining individually suggestive, but nonsignificant, blood RNA levels were significantly predictive and may improve diagnostic accuracy for Alzheimer’s disease. Therefore, we propose that patient features that do not individually predict cognitive status might still contribute to overall cognitive decline through interactions that can be elucidated through machine learning.

## 1. Introduction

Late-onset Alzheimer’s disease (AD) has long devastated the elderly population, affecting over 10% of adults older than 65 [1]. While AD was once considered a discrete disease with a single phenotype, the National Institute on Aging and Alzheimer’s Association now classifies AD as a continuum of biomarker and neuroimaging levels under a biological construct [2], indicating that biology and cognitive decline are intertwined. Although many techniques are available to diagnose cognitive decline, undetected dementia remains at 55–68% globally [3]. Patients are often unaware of their cognitive decline [4], limiting their ability to adequately address physical and mental limitations caused by dementia. Furthermore, 15–35% of patients older than 65 who are offered cognitive screening refuse to perform cognitive assessments, especially if they do not personally know anyone affected with AD [5,6]. Even after being referred by a community pharmacist to a physician for a follow-up cognitive study, almost 80% of pre-screened patients did not see a physician within 60 days, and over 40% of patients were unwilling to pay for additional cognitive screening [7]. Older adults often view cognitive assessments as embarrassing, invasive, and confusing [8,9]. However, without a proper diagnosis, patients may postpone end-of-life planning until their memory further deteriorates, or they may be incapable of completing an advance directive (i.e., living will) if their memory has already deteriorated [10]. Therefore, it is imperative that cognitive function is accurately and affordably diagnosed early in disease progression in a manner that makes the patient feel comfortable.

The Clinical Dementia Rating (CDR) [11] is a widely used cognitive diagnostic assessment [12] that is comparable to the gold standard dementia diagnostic criteria [13]. The CDR examination is complex and requires 8–9 h of training before it can be administered by a researcher or clinician. Additionally, the patient examination is cumbersome and takes between 30–90 min [14], requiring an assessment of differences in patient memory compared to the memory from a reliable informant (e.g., family member or caregiver) [15]. In contrast, a blood draw is less intrusive and could be completed in a matter of minutes by healthcare professionals without specialized cognitive training. Therefore, a blood draw establishing the biological basis for cognitive decline would improve diagnostic accessibility and potentially increase patient response rate.

Previous studies have used blood mRNA levels to predict cognitive decline with mixed results. Lee and Lee [16] identified significant differences in expression in genes that were enriched in inflammatory and immune pathways, although the predictive power varied significantly between studies. Similarly, differential expression in genes implicated in other autoimmune diseases have previously been linked to AD [17]. RNA levels are also distinct across cognitive status, independent of white matter hyperintensities, which indicates that RNA levels alone may be sufficient as a diagnostic technique [18]. Other studies have aimed to use peripheral blood to predict cognitive decline before symptoms become evident, and increases in extracellular RNA in blood plasma are associated with later developing AD [19]. Here, we used blood RNA levels in the Alzheimer’s Disease Neuroimaging Initiative (ADNI) dataset to predict patient CDR. We performed dimensionality reduction to avoid overfitting on the training dataset by using an analysis of variance (ANOVA), which is a statistical method that analyzes differences between group means in a sample, as an initial filter to select the most significant microarray probes, similar to other machine learning analyses of microarray data [20]. We filtered 49,386 RNA probes based on their ANOVA *p*-values, and we used various *p*-value thresholds to assess predictive accuracy. Probes exceeding the *p*-value threshold were used as input features in a support vector machine to predict CDR scores for 741 participants in the ADNI dataset.

## 2. Materials and Methods

Data used in the preparation of this article were obtained from the Alzheimer’s Disease Neuroimaging Initiative (ADNI) database. The ADNI was launched in 2003 as a public-private partnership, led by Principal Investigator Michael W. Weiner, MD. The primary goal of ADNI has been to test whether serial magnetic resonance imaging (MRI), positron emission tomography (PET), other biological markers, and clinical and neuropsychological assessment can be combined to measure the progression of mild cognitive impairment (MCI) and early Alzheimer’s disease (AD).

We used RNA expression data from an Affymetrix HG U219 Array (Affymetrix, Santa Clara, California, USA). ADNI preprocessed the raw expression values using the Robust Multi-chip Average (RMA) normalization method before mapping and annotating the probe sets to the hg19 human reference genome. The ADNI Genetics Core performed several other quality control measures on the dataset. Array plate randomization, gender and diagnosis balance, participant and probe quality control, and SNP-transcript cis-eQTL posterior probabilities were completed to ensure that analyses conducted on the dataset are not impacted by confounding factors. We also ensured that each individual had taken a CDR exam, which limited the available dataset to 49,386 probes across 741 participants whose cognitive abilities ranged from normal to severe dementia.

We labelled participants in one of three cognitive groups based on their most recent CDR score: cognitive normal (CDR = 0), mild cognitive impairment (CDR = 0.5), and probable AD (CDR ≥ 1.0). We clustered CDR levels of 2.0 and 3.0 into the probable AD group to maintain predictive power because only 15 individuals had a CDR score of 2.0 and only one individual had a CDR score of 3.0. In total, 250 individuals were cognitive normal, 411 individuals had mild cognitive impairment, and 80 individuals had probable AD based on their respective CDR score.

We conducted a one-way analysis of variance (ANOVA) on each of the 49,386 probes individually to test the extent to which expression levels for each RNA probe significantly differed between the three groups. After a Bonferroni correction, our significance threshold was 1.012 × 10^−6^. We further assessed sex-specific biases in the three groups using the five X-inactive specific transcript (XIST) probes in the dataset, and we determined that no significant sex differences exist between the three cognitive groups (*p*-values = 0.0145, 0.017, 0.019, 0.041, and 0.068).

**Figure 1:**
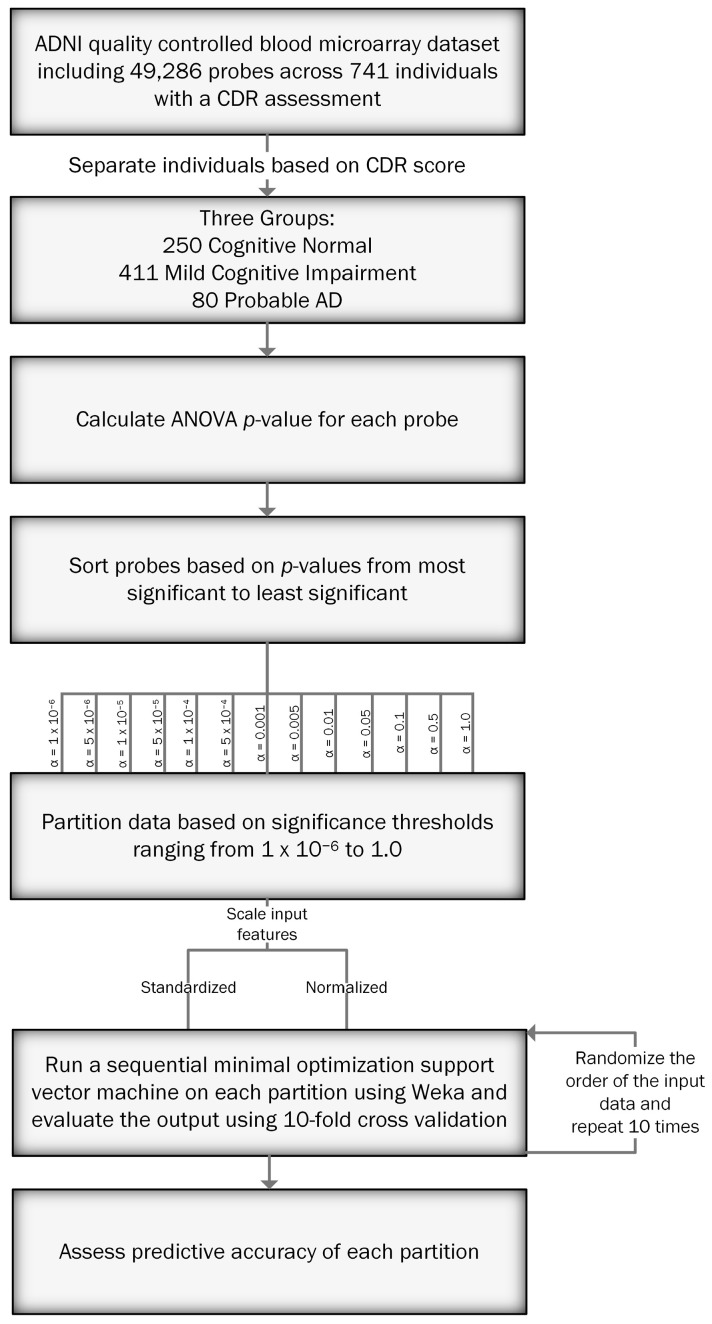
Flowchart depicting the analysis process to predict cognitive status using the ADNI blood microarray dataset.

We then pruned our dataset based on the following α values: 1.0 × 10^−6^, 5.0 × 10^−6^, 1.0 × 10^−5^, 5.0 × 10^−5^, 1.0 × 10^−4^, 5.0 × 10^−4^, 0.001, 0.005, 0.01, 0.05, 0.1, 0.5, 1.0. These cutoff criteria were used for feature selection of RNA probes as input in a machine learning model. We used the Waikato Environment for Knowledge Analysis (Weka) [21] implementation of sequential minimal optimization, which is a fast heuristic of a polynomial kernel support vector machine. Support vector machines are non-probabilistic binary linear classifiers that separate training data so that the separator creates the largest gap possible between different groups. We scaled our input features in two ways: 1) normalizing input features by rescaling the data between 0 and 1, and 2) standardizing input features based on standard deviations from the sample mean. All other hyperparameters were left at their default settings in Weka. We performed 10-fold cross validation for each α value and scaled input values, repeating each analysis 10 times by randomizing the seed used for 10-fold cross validation to limit the potential effects of training set splitting on our prediction. We then assessed the predictive accuracy of each partition. Figure 1 depicts the process used to analyze the data.

Additionally, we assessed the merit of our machine learning results through using random permutations. By randomly permuting the output classes on the training set with the highest predictive accuracy (i.e., using the α value with the highest predictive accuracy and maintaining the same number of training features in the permutations), we were able to assess algorithmic bias inherent in the dataset and calculate a *p*-value for the results from our model. We calculated the predictive accuracy, precision, recall, and Receiver operating characteristic (ROC) curve area for 100 separate permutations using the same input parameters as our predictive model: a polynomial kernel support vector machine in Weka. We then calculated the mean and standard deviation across all permutations to assess the accuracy of our predictive model using the correct input labels.

## 3. Results

We first analyzed each probe individually to determine if significant differences in RNA levels exist between the three cognitive groups. All three Chloride Intracellular Channel 1 (*CLIC1*) probes were in the top five most significant probes for the dataset, with one probe exceeding the Bonferroni threshold for significance. Table 1 shows the mean expression levels for the significant *CLIC1* probe (11757474_x_at). The mean levels for the cognitive normal and mild cognitive impairment groups do not significantly differ (*p*-value = 0.30). However, the probable AD group has a significantly higher mean expression than the other two groups (*p*-value = 5.2781 × 10^−7^) and has a moderate to large effect size (Cohen’s d = 0.6295).

**Figure 2:**
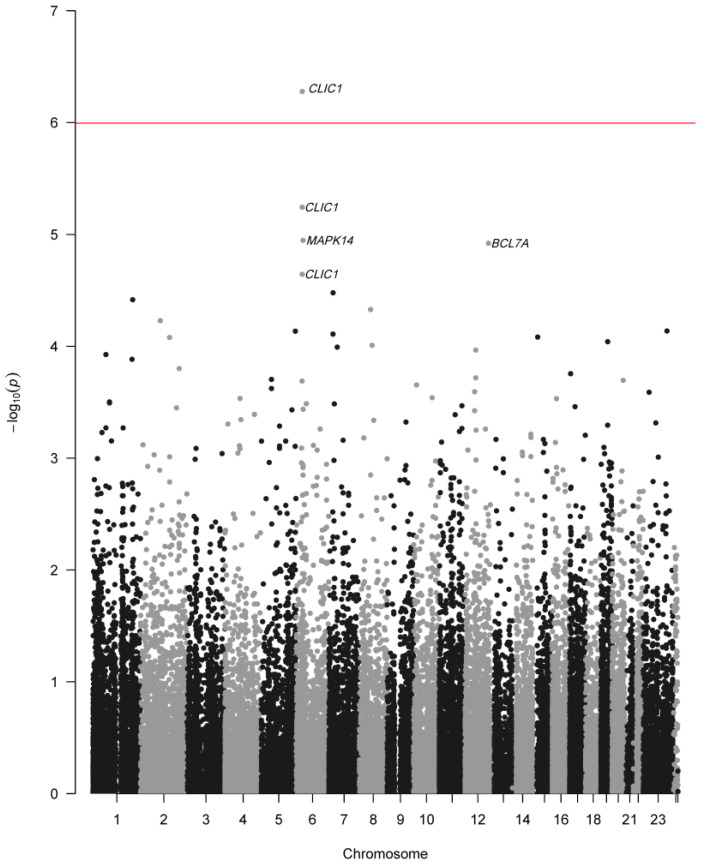
ANOVA p-values for 49,386 Microarray Probes. Negative log-transformed *p*-values for each probe, sorted by chromosome position. Black and grey coloring alternate to indicate different chromosomes. The dashed line shows the Bonferroni threshold for significance after correcting for multiple tests. Only one probe for the Chloride Intracellular Channel 1 (CLIC1) exceeded the significance threshold.

Although mean RNA levels for *CLIC1* probe 11757474_x_at statistically differ from mean RNA levels in the other two cognitive groups, that probe alone is insufficient to predict cognitive status. Therefore, we examined combinations of individually nonsignificant probes to predict CDR levels. Collectively, the probes roughly followed expected significance values, with about 5% of the probes having *p*-values less than or equal to 0.05. Figure 2 depicts the ANOVA *p*-values for each probe and the Bonferroni corrected α value for the dataset.

**Figure 3:**
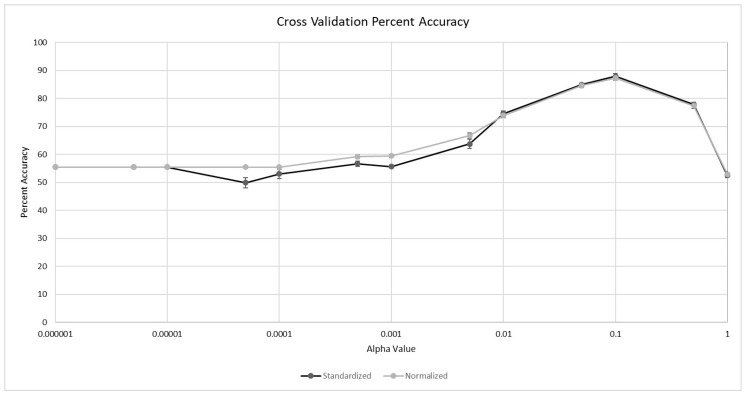
Percent accuracy for the support vector machines. Accuracies for predictions using both the standardized and normalized datasets are plotted. The α values are the criteria for feature selection for each model and are shown on the *X*-axis.

We used 13 α values for feature selection to assess the combined predictive power of individually nonsignificant probes ranging from 1.0 × 10^−6^ (one probe) to 1.0 (all probes). Probes with *p*-values less than or equal to the selected α value were either standardized or normalized and included in two separate models. We tested the effects of standardization and normalization to ensure that our analyses were not affected by the assumption of a Gaussian distribution. Additionally, since 10-fold cross validation is also subject to biases related to splits when the dataset is relatively small, we permutated the input files 10 times and performed 10-fold cross validation on each permutation to calculate a standard deviation for the support vector machine on each α value. Figure 3 shows the percent accuracies and standard deviations in predicting cognitive status for each α value using 10-fold cross validation. The highest predictive power occurred when using a α of 0.1. The standardized permutations had a mean accuracy of 87.87% (s = 1.02), while the normalized permutations had a mean accuracy of 87.25% (s = 0.77). A *t*-test showed a significant difference between the maximum percent accuracies between the normalized and standardized datasets (*p*-value = 5.039 × 10^−38^), although the difference in the mean predictive accuracy was minimal (0.52%). All ten permutations in both datasets had a 0% false positive rate for AD. The confusion matrix for the most accurate prediction from 10-fold cross validation of the standardized data with a α of 0.1 is shown in Table 2. The overall precision, recall, and ROC curve area for the model was 0.902, 0.895, and 0.904, respectively. Using additional probes with *p*-values higher than 0.1 significantly decreased the predictive accuracy, eventually leading to the baseline accuracy of 55.46%.

We performed 100 random permutations of the training labels starting with the dataset that was created using 0.1 as a α value for feature selection because it had the greatest predictive accuracy. We found that 10-fold cross validation of the support vector machine of the dataset with the CDR labels from ADNI significantly differs from the 10-fold cross validation of the null randomized dataset. The average precision, recall, and ROC curve area for the random permutations was 0.434 ± 0.180, 0.483 ± 0.0172, and 0.502 ± 0.0166, respectively. Our best model using the true CDR labels outperformed the mean precision, accuracy, and ROC curve area of the random permutations by 0.468, 0.412, and 0.402, respectively. Additionally, the highest random permutation reported precision, accuracy, and ROC curve area of only 0.480, 0.533, and 0.550, respectively. Please specify whether linear or kernel SVM was used.

## 4. Discussion

We identified one significant probe in the chloride intracellular channel 1 (*CLIC1*). *CLIC1* has previously been linked to AD and induces neurotoxin production in the presence of β-amyloid (Aβ) protein [22]. A direct link between *CLIC1* expression and Aβ-induced microglial activation has also been established [23]. Our analyses show that significantly higher levels of *CLIC1* exist in AD patients compared with cognitive normal and mild cognitive impairment groups and the effect size of the difference in moderate to high. These results support previous indications that *CLICL1* levels increase in AD patients and additionally show that these differences are detectable in peripheral blood. However, *CLIC1* expression alone is insufficient to accurately diagnose cognitive status in an individual with AD.

Additionally, B-cell CLL/lymphoma 7 protein family member A (*BCL7A*) and Mitogen-Activated Protein Kinase 14 (*MAPK14*) individually approached significance in our dataset. Although *BCL7A* has not previously been directly linked to AD, it is known that B cells are impaired throughout the aging process, which likely compromises the immune system [24], and a compromised peripheral immune system is linked to AD [25]. *MAPK14* has previously been used as a therapeutic target of AD to regulate inflammation and target innate immune brain responses [26]. *MAPK14* regulates immunological responses and integral in the production of chemokines and cytokines in astrocytes [27]. Both genes are involved in immune response, and support previous research indicating association of AD with differential expression in gene integral to the immune system [16]. Additionally, *MAPK14* is located 4 Mbp downstream from *CLIC1* on chromosome 6, and the proximity to *CLIC1* may cause a false positive significant *p*-value due to gene interactions or linkage disequilibrium. However, our analyses also show that these genes alone are not sufficient to predict AD status.

## 5. Conclusions

Our analyses indicate that machine learning may be able to predict cognitive decline in individuals using RNA levels from a blood microarray by taking into account small differences in expression that are individually nonsignificant. A support vector machine was able to increase predictive accuracy of AD from a 55% baseline to almost 90%. There was also a clear directionality in the predictions, with incorrect predictions for cognitive normal and AD patients more likely to be one cognitive group away from the diagnosis (e.g., incorrect predictions for AD patients were more likely to be predicted as mild cognitive impairment than cognitive normal). This directionality indicates that blood RNA levels gradually change as a patient progresses from a cognitive normal state to AD and supports the National Institute on Aging and Alzheimer’s Association’s guidelines that label AD on a continuum.

Our analyses also suggest that combining individually nonsignificant traits that suggest an association (e.g., *p*-value less than 0.1) may increase the accuracy of disease assessments and be a viable method of feature selection. Therefore, we propose that using a similar technique to combine other biomarkers in machine learning models may further increase the accuracy of early AD diagnoses even when those traits alone are insufficient to predict cognitive status. At the population level, low body mass index [28], vital exhaustion [29], and changes in retinal microvasculature [30] each indicate early signs of Alzheimer’s disease. However, the natural variance within the population limits the use of these biomarkers in a clinical setting. Similarly, individual RNA probes within the ADNI dataset have reported levels that significantly overlap between cognitive groups and cannot be used in isolation to diagnose a patient. However, predictions became much more accurate when considering thousands of minor differences in RNA levels. Similarly, machine learning may be able to combine minor, individually nonsignificant, differences across diverse biomarkers to improve predictive accuracy for AD diagnosis in the future.

## Figures and Tables

**Table 1:** Mean RNA expression levels for the 11757474_x_at probe. The three groups are based on Clinical Dementia Rating (CDR): cognitive normal (CDR = 0), mild cognitive impairment (CDR = 0.5), and Alzheimer’s disease (CDR ≥ 1.0). The mean RNA levels for the Alzheimer’s disease group significantly differs from the other two groups.

Group	N	Mean 11757474_x_at Expression	Standard Deviation 11757474_x_at Expression	Standard Error 11757474_x_at Expression	Percent Female (%)	Average Age ± One Standard Deviation
**Cognitive Normal**	250	11.574	0.1104	0.007	52.21	76.28 ± 6.46
**Mild Cognitive Impairment**	411	11.583	0.1069	0.0053	41.95	73.39 ± 7.94
**Alzheimer’s Disease**	80	11.6479	0.1088	0.0122	39.76	77.75 ± 8.43

**Table 2:** Confusion matrix of the best cross validation using standardization with a α of 0.1. The model prediction and the CDR assessment for all three cognitive groups is shown. Additionally, precision, accuracy, and ROC curve area, including the weighted average for the model, are depicted.

Model Prediction	Cognitive Normal (CDR = 0)	Mild Cognitive Impairment (CDR = 0.5)	Probable Alzheimer’s Disease (CDR ≥ 1.0)	Precision	Recall	Receiver Operating Characteristic (ROC) Curve Area
CDR Assessment						
**Cognitive Normal**	214	36	0	0.926	0.856	0.921
**Mild Cognitive Impairment**	12	399	0	0.867	0.971	0.894
**Probable Alzheimer’s Disease**	5	25	50	1.0	0.625	0.906
**Weighted Average**	N/A	N/A	N/A	0.902	0.895	0.904
